# Heart Rate and Cardiovascular Responses to Commercial Flights: Relationships with Physical Fitness

**DOI:** 10.3389/fphys.2016.00648

**Published:** 2016-12-27

**Authors:** Iransé Oliveira-Silva, Anthony S. Leicht, Milton R. Moraes, Herbert G. Simões, Sebastián Del Rosso, Cláudio Córdova, Daniel A. Boullosa

**Affiliations:** ^1^Post-Graduate Program in Physical Education, Catholic University of BrasiliaÁguas Claras, Brazil; ^2^Physical Education, UniEVANGÉLICA – Centro UniversitárioAnápolis, Brazil; ^3^Sport and Exercise Science, College of Healthcare Sciences, James Cook UniversityTownsville, QLD, Australia

**Keywords:** autonomic nervous system, blood pressure, cardiovascular reactivity, cardiovascular stress response, physical activity, exercise

## Abstract

The aim of this study was to examine the influence of physical fitness on cardiac autonomic control in passengers prior to, during and following commercial flights. Twenty-two, physically active men (36.4 ± 6.4 years) undertook assessments of physical fitness followed by recordings of 24-h heart rate (HR), heart rate variability (HRV), and blood pressure (BP) on a Control (no flight) and Experimental (flight) day. Recordings were analyzed using a two-way analysis of variance for repeated measures with relationships between variables examined via Pearson product-moment correlation coefficients. Compared to the Control day, 24-h HR was significantly greater (>7%) and HRV measures (5–39%) significantly lower on the Experimental day. During the 1-h flight, HR (24%), and BP (6%) were increased while measures of HRV (26–45%) were reduced. Absolute values of HRV during the Experimental day and relative changes in HRV measures (Control-Experimental) were significantly correlated with measures of aerobic fitness (*r* = 0.43 to 0.51; −0.53 to −0.52) and body composition (*r* = −0.63 to −0.43; 0.48–0.61). The current results demonstrated that short-term commercial flying significantly altered cardiovascular function including the reduction of parasympathetic modulations. Further, greater physical fitness and lower body fat composition were associated with greater cardiac autonomic control for passengers during flights. Enhanced physical fitness and leaner body composition may enable passengers to cope better with the cardiovascular stress and high allostatic load associated with air travel for enhanced passenger well-being.

## Introduction

Commercial flights are currently used worldwide by more than 2.7 million people annually for business and tourism purposes (Peterson et al., 2013). Besides its efficacy and relative comfort, commercial flights have been considered among the safest modes of transportation (Norbäck et al., 2006). Previously, stressful factors inherent to flights such as increased anxiety during take-off and landing, changes in body position induced by aircraft acceleration and deceleration, aircraft noise, and fluctuations in the aircraft's cabin pressure have been demonstrated to interfere in the well-being and mood state of the crew (Chandra and Conry, 2013). These flight experiences along with the stress of daily living situations such as those related to work, may lead travelers to endure a high allostatic load (McEwen and Seeman, 1999; Tonello et al., 2014). Repeated exposure to a high allostatic load could be associated with physiological imbalances leading to health threatening risk factors (e.g., inflammatory process, hostility, depression; McEwen and Seeman, 1999; Olson et al., 2015). Therefore, monitoring allostatic load and improving allostatic tolerance may be vital for health and well-being.

Allostatic capacity can be monitored by the activity of the autonomic nervous system (ANS) through its regulation of the cardiovascular, respiratory and neuroendocrine systems (Verlato et al., 2011). The analysis of cardiovascular control, termed heart rate variability (HRV), has been as reported as an effective tool to identify changes in ANS activity (Task Force, 1996) in response to different stressors (Teisala et al., 2014). In addition to traditional HRV measures of time and frequency domains, other non-linear measures of HR dynamics have been suggested to provide useful prognostic information in various clinical settings while their reproducibility may be better than that of traditional indices (Huikuri et al., 2009). Consideration of both linear and non-linear HRV measures could be especially important during ambulatory conditions in which the validity and reproducibility of these measures could be different depending on different factors as physical activity levels or sample characteristics (Hautala et al., 2010; Tonello et al., 2015). Previous studies with military pilots demonstrated important HRV and autonomic adjustments during flights (Dussault et al., 2009; Oliveira-Silva and Boullosa, 2015). These flight-induced autonomic changes may trigger several physiological adjustments to maintain homeostasis during a potentially detrimental activity (McEwen and Seeman, 1999). It is important to point out that the reported incidence of medical emergencies during civil aviation is low with a rate of 1 per 604 flights or ~0.002% (Peterson et al., 2013). Despite this low rate, some individuals experience difficulties during aviation with the most prevalent problems being: syncope (37.4%), respiratory distress (e.g., dyspnea, 12.1%), and nausea (9.5%) (Peterson et al., 2013). As a result of these difficulties, Limper et al. (2014) expressed the need for medical screening of individuals before flights and the importance of maintaining satisfactory health in order to cope with gravitational and pressure changes associated with commercial flights.

Recent medical guidelines for air travel have emphasized the necessity to consider different aspects of passengers' health or medical state (Thibeault et al., 2015), however these guidelines do not address the role of physical fitness for passenger well-being during commercial flights. Typically, these air travel guidelines encompass physical and psychological issues regarding specific health-related problems and diseases rather than generic well-being (Aerospace Medical Association Medical Guidelines Task, 2003; Thibeault et al., 2015). Consideration of other important health factors such as physical fitness or physiological function like ANS activity though are rarely considered important for air travel (Oliveira-Silva and Boullosa, 2015). Individuals with a greater physical fitness have been reported to demonstrate an increased parasympathetic modulation at rest (Hautala et al., 2010), a better ANS modulation during stressful situations (Teisala et al., 2014), and reduced risk of future cardiac events (Fu and Longhurst, 2009). Recently Oliveira-Silva and Boullosa (2015) demonstrated that military pilots with better physical fitness (i.e., higher aerobic capacity and lower body fatness) exhibited a greater cardiac autonomic control (i.e., HR complexity) during training flights when compared to a control day which highlighted physical fitness as an important factor to counteract the effects of stress.

While physical fitness parameters have been considered important for managing physiological responses to stress, to the best of our knowledge, there have been no studies examining the possible role of physical fitness in regulating cardiovascular stress induced by commercial flights, a common and growing activity experienced by millions of people worldwide each year. Thus, the purpose of the present study was to examine the influence of physical fitness on the autonomic modulation of HR and blood pressure (BP) responses of passengers during commercial flights. Our hypothesis was that greater physical fitness (e.g., greater aerobic capacity and muscular strength, and lower % body fat) would result in better cardiovascular control (HRV and BP) for passengers during flights. Greater cardiovascular control and HRV via enhanced physical fitness may enable passengers to cope better with the physiological stresses associated with air travel and maximize passenger well-being.

## Methods

### Participants

Twenty-two physically active men, free from pathological conditions (i.e., diabetes, hypertension, cardiovascular disease, depression, etc.) and medications that could interfere with the outcome measures, volunteered for participation in this study. Their mean ± standard deviation (SD) age, body mass, height, body mass index (BMI), and percentage of body fat was: 36.4 ± 6.4 years, 77.5 ± 12.0 kg, 1.76 ± 0.5 m, 25.1 ± 3.6 kg/m^2^, and 17.7 ± 5.1%, respectively. All participants had previous experience with flying but had accumulated < 50 flying hours as a passenger on commercial airlines. Prior to participation and after discussion of the procedures, participants signed a consent form in accordance with the approved protocol (135.530) by the local Ethics Committee of Catholic University of Brasilia in accordance with the Declaration of Helsinki.

### Procedures

Data recordings were performed prior to, during and following travel between the cities of Anápolis, Goiβnia, Brasília, Belo Horizonte, and São Paulo in Brazil, between the months of August and October of 2014 (i.e., spring). The outdoor ambient temperature ranged between 14 and 28°C with an air relative humidity of 27–78%. Participants were initially assessed in Anápolis as most resided there and traveled to airports by car (< 2 h) prior to undertaking commercial flights from Goiβnia to São Paulo or Belo Horizonte, and from Brasília to São Paulo. Each commercial flight lasted an average of 1 h and started between 15:00 and 17:00.

**Figure 1:**
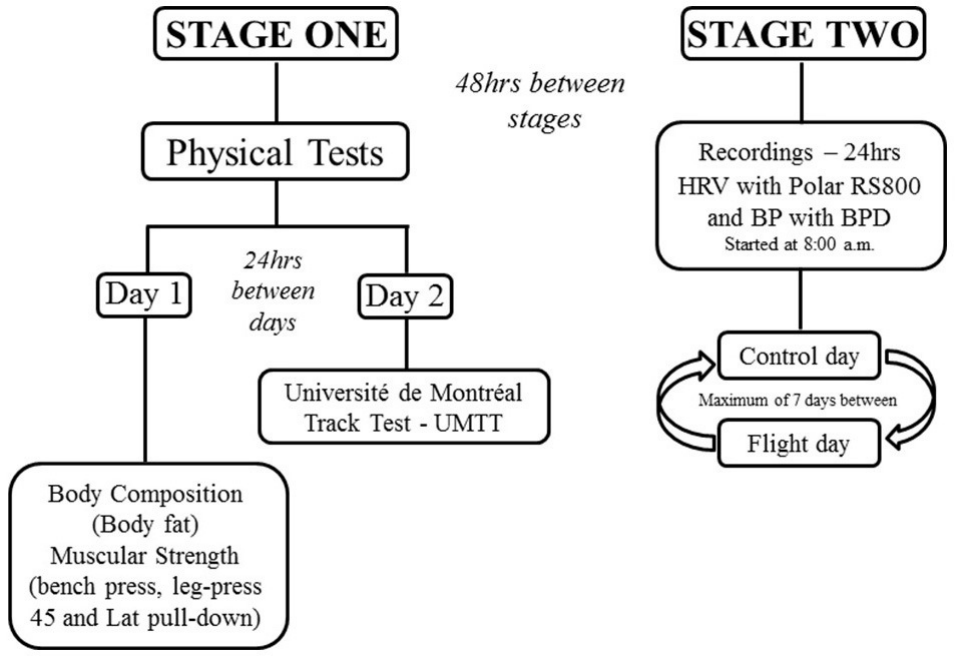
**Experimental design**.

All participants undertook initial assessments of physical fitness (Stage 1) followed by assessment of 24-h HRV prior to and following commercial flights (Stage 2, Figure 1). All physical fitness tests were performed between 08:00 and 10:00 on two separate days, 24-h apart, in the week before undertaking the commercial flight. On the first day, participants were evaluated for body composition and muscular strength (e.g., bench press, leg-press 45° and lat pull-down). On the second day, participants completed the “*Université de Montréal Track Test*” (UMTT) (Léger and Boucher, 1980) to assess aerobic running capacity. Forty-eight hours later, participants undertook an evaluation of the autonomic control of HR and BP during a control day (without flight) and a flight day (flight ~ 1hr) in a randomized order. Heart rate and BP recordings (24-h) started and ended at 08:00 a.m. The time interval for physical tests and HRV and BP recordings was 7–10 days (Figure 1).

### Body composition

Body mass was assessed via calibrated scales (PL 200, Filizola, Brazil) while height was assessed via a stadiometer (Sanny, Brazil). Skinfold thicknesses were assessed using a skinfold caliper (Langue, Cambridge Scientific Industries, USA) and body fatness (%) estimated using previously validated equations (Jackson and Pollock, 1978).

### Muscular strength

Muscular strength was evaluated during a bench press, leg press at 45° and lat pull-down exercise on specialized exercise machines (Technogym, Italy). All participants were familiar with these exercises and had more than 1 year of regular, resistance exercise training in the gym. The equipment was adjusted appropriately to each participant to ensure optimal body and joint positioning. Participants performed a one-repetition maximum (1-RM) protocol with the 1-RM score recorded as the highest weight successfully lifted with proper technique (Garber et al., 2011).

### Aerobic capacity

Aerobic capacity was evaluated with the “*Université de Montréal Track Test”* (UMTT) (Léger and Boucher, 1980) for determination of maximal aerobic speed (MAS), and subsequent estimation of maximum oxygen uptake (VO_2_max) [MAS × 3.5 ml·kg^−1^·min^−1^] (Léger and Mercier, 1984). The test was performed on a 400 m track with individuals following a cyclist that reproduced the original protocol as previously described (Boullosa and Tuimil, 2009). Briefly, the protocol started at 7 km·h^−1^ with increments of 1 km·h^−1^ every 2 min until participants could not maintain the velocity imposed by the cyclist. The time to complete the UMTT (T_UMTT_) was also recorded (Boullosa and Tuimil, 2009).

### Heart rate variability

At 48 h after the completion of the physical fitness tests, two R-R recordings separated by at least 72 h were obtained with a HR monitor (RS800, Polar Electro Oy, Finland) that has been previously validated (Wallén et al., 2012). These recordings were conducted on a control (no flight) and experimental (flight) day with the order of days randomly assigned. During the control day, participants undertook their normal daily activities and did not perform any exercise. The experimental day consisted of normal daily activities without exercise and a commercial flight (64:21 ± 05:42 mins:secs) in the afternoon (15:00–17:00).

The HR recording commenced at 08:00 a.m. in the presence of the primary researcher. During the experimental day, participants were advised to trigger the “lap” function of the HR monitor at four moments to identify specific travel activities for standardization: (i) before passing through the metal detector at the airport; (ii) after passing through the airport metal detector and restoring the equipment (HR and BP); (iii) when the aircraft initiated its take-off maneuver; and (iv) when the aircraft completed its taxi process at the destination airport. Each flight covered a mean distance of ~900 km at an altitude of ~11,000 m. Heart rate recordings were visually inspected and manually filtered within specialized software (Polar Pro Trainer, v 5.35.161, Polar Electro Oy, Finland), and subsequently analyzed by dedicated HRV analysis software (HRV Kubios v2.0, Kuopio University, Finland). These recordings were also divided into1-h blocks and visually inspected to identify those close to the flight (i.e., 1-h blocks from 2 h before until 2 h after the flight). For comparisons between days for each participant, similar timings were used for the control day (e.g., 15:30–16:30 on the control day was compared with the 15:30–16:30 flight time on the experimental day).

The HR and HRV parameters selected for analysis were: heart rate (HR), standard deviation of the RR series (SDNN), square root of the mean of the sum of the squares of differences between adjacent RR intervals (RMSSD), standard deviation of the instantaneous RR intervals of the Poincaré plot (SD1), the standard deviation of RR intervals analyzed over the long-term of the Poincaré plot (SD2), sample entropy (SampEn), exponent of short-term fractal scaling (α1), exponent of long-term fractal scaling (α2), and the spectral components of the low frequency (LF, 0.04–0.15 Hz) and high frequency (HF, 0.015–0.4 Hz) bands, as previously examined (Task Force, 1996; Boullosa et al., 2014).

### Blood pressure and rate pressure product

Blood pressure was recorded with a blood pressure monitoring device (BPD) (Dyna Mapa - version 12, Cardios®, Brazil) approved by the British Society (Jones et al., 2000), at the same times as the HR recordings. Following standardized procedures (Sociedade Brasileira Cardiologia, 2011), BP was recorded every 30 min with recordings exported to a computer for later analysis by the corresponding software (BP3MZ1, Onbo Electronic, China). Recordings of systolic (SBP), diastolic (DBP) and mean BP [0.333 × (SBP-DBP) + DBP, MBP] were averaged to produce hourly measures as well as the average of the entire 24 h, similar to the HRV measures. Additionally, the rate pressure product (RPP), a measure of cardiac myocardial workload and the product of the hourly average SBP and mean HR, was calculated each hour and for the entire day (24-h).

### Statistical analysis

Statistical analyses were performed using a statistical package (SPSS, v 20.0, IBM, Chicago, USA) with descriptive data presented as mean ± SD and confidence limits (90%). Normality was tested using the Kolmogorov-Smirnov test with Lilliefors correction. Variables with a non-normal distribution were log-transformed (Ln) for analysis. Differences (%) between the control day and experimental day for each of the described periods were calculated as follows: (control day - experimental day) / control day × 100. The differences between the parameters at specific times (e.g., 2 h before flight, 1 h before the flight, 1 h during the flight, 1 h after the flight, 2 h after the flight) and between days (i.e., control and experimental) were examined using a two-way analysis of variance (ANOVA) for repeated measures (days × times) and *post-hoc* paired comparisons with Bonferroni correction. The magnitude of the changes between days was assessed by the effect size (ES) using Cohen's *d* (Sullivan and Feinn, 2012) where *d* = 0.01–0.19 considered trivial, 0.20–0.49 considered small, *d* = 0.50–0.79 considered medium, and *d* > 0.79 large. To examine the relationship between physical fitness and HRV (absolute values and absolute and relative differences in HRV—control vs. experimental), *Pearson* product-moment correlation coefficients (r) were calculated. The level of significance was set at *p* < 0.05.

## Results

Table 1 depicts the mean values for the physical tests performed by the participants.

**Table 1:** **Physical test performances**.

**Variables**	**Mean**	**90% CL**
Bench press (kg)	67.9±13.5	63.1–72.6
Lat pull-down (kg)	66.1±6.8	63.7–68.5
Leg Press 45° (kg)	167.7±32.5	156.3–179.1
Muscle strength (kg)	100.6±16.5	94.8–106.3
MAS (km·h^−1^)	12.7±2.8	11.8–13.7
T_UMTT_ (s)	842.7±223.5	719.4–947.8
VO_2_max(mL·kg^−1^· min^−1^)	44.7±9.7	41.2–48.1

*Muscular strength: average of the three exercises (bench press, lat pull down, and leg press 45°); MAS, maximum aerobic speed; T_UMTT_, Time to complete the Université de Montréal Track Test; VO_2_max, estimated maximum oxygen consumption; CL: confidence limits*.

Twenty-four hours HR and HRV results during the control and experimental days are shown in Table 2. All variables were similar between days except for a significantly greater HR, and significantly lower LnHF and α_2_ for the experimental day (ES = 0.49–1.01, Table 2).

**Table 2:** **Heart rate and heart rate variability measures derived from 24-h recordings during control and experimental days**.

			**Magnitude of changes between days**		
	**Control day**	**Experimental day**	**p**	**Δ%;%90 CL**	**ES**
HR (bpm)	71.9±8.0	78.8±11.7^a^	0.03	7.4;±4.1	0.49
RMSSD (ms)	41.8±16.9	41.5±16.7	0.95	−0.7;±8.1	0.01
SDNN (ms)	96.9±26.1	93.7±25.3	0.62	−3.2;±10.5	0.12
LnLF (ms^2^)	7.3±0.6	7.2±0.5	0.91	−8.0;±618	0.03
LnHF (ms^2^)	6.2±0.8	5.9±0.8^a^	0.02	39.2;±567	1.01
SD1 (ms)	29.9±12.5	29.4±11.9	0.90	−1.4;±5.8	0.03
SD2 (ms)	132.9±34.8	128.7±34.3	0.60	−3.2;±13.9	0.12
SampEn	1.01±0.09	0.96±0.18	0.17	−5.1;±0.06	0.35
α1	1.35±0.14	1.34±0.16	0.81	−0.6;±0.06	0.06
α2	0.99±0.08	0.93±0.08^a^	0.00	−5.8;±0.03	0.75

*HR, heart rate; RMSSD, root mean square of successive differences between normal sinus R-R intervals; SDNN, standard deviation of all R-R intervals; LnLF, natural log of the Low Frequency component; LnHF, natural log of the High Frequency component; SD1, short-term beat-to-beat R-R variability from the Poincaré plot; SD2, long-term beat-to-beat variability from the Poincaré plot; SampEn, sample entropy; α1, detrended fluctuations of short-term fractal scaling; α2, detrended fluctuations of long-term fractal scaling. %Δ: percentage difference between days; CL: confidence limits; ES: effect size*;

a *: p < 0.05 vs. Control day*.

Linear and non-linear HRV measures (1-h recordings) prior to, during and after the flight on the experimental and control days are shown in Tables 3, 4, respectively. Compared to the Control day, HR was significantly greater (>12%) prior to, during and after the flight on the Experimental day (ES = 0.58–1.13, Table 3). In contrast, RMSSD was significantly lower (>20%) in the hour prior to, during and the immediate hour after the flight compared to the control day (ES = 0.42–0.95, Table 3). During the flight, LnHF was significantly lower (>25%, ES = 0.94) compared to the control day while SDNN (< 15%) and LnLF (< 7%) were similar between days (ES = 0.34–0.52, Table 3).

**Table 3:** **Linear HRV measures (~1-h recordings) before, during and after the flight on the experimental day and at matched times on the control day**.

	**Moment**	**Control day**	**Experimental day**	**Magnitude of changes between days**	
				**%Δ; ± 90% CL**	**ES**
HR (bpm)	2-h before	74±15	84±16^a^	13.7;±5.6	0.64
	1-h before	74±17	83±14^a^	12.3;±5.6	0.58
	Flight	72±13	89±17^a^	24.1;±5.5	1.13
	1-h after	73±16	82±16	12.5;±5.7	0.57
	2-h after	71±14	83±15^a^	17.0;±5.3	0.80
RMSSD (ms)	2-h before	42.1±24.5	35.5±20.7	−15.6;±8.1	0.29
	1-h before	44.0±33.1	30.6±16.9^a^	−30.4;±9.1	0.42
	Flight	44.9±25.4	24.9±15.0^a^^,^^b^	−44.5;±7.5	0.95
	1-h after	41.9±21.4	33.3±16.6^a^	−20.1;±6.8	0.45
	2-h after	46.1±25.6	36.8±20.3	−10.8;±8.2	0.40
SDNN (ms)	2-h before	95.8±42.6	85.4±43.9	−10.8;±15.5	0.24
	1-h before	97.6±46.6	82.6±32.1	−15.3;±14.3	0.37
	Flight	95.6±44.4	81.9±34.2	−14.3;±14.2	0.34
	1-h after	99.6±56.3	81.8±30.1	−17.8;±16.1	0.39
	2-h after	97.8±38.4	85.2±31.6	−12.8;±12.6	0.35
LnLF (ms^2^)	2-h before	3.1±0.3	2.9±0.3	−4.2;±14.3	0.35
	1-h before	3.1±0.3	3.0±0.3	−4.4;±17.1	0.28
	Flight	3.0±0.3	2.9±0.3	−6.6;±15.8	0.52
	1-h after	3.0±0.3	3.0±0.3	−2.8;±16.2	0.17
	2-h after	3.1±0.3	3.1±0.3	−2.4;±17.5	0.06
LnHF (ms^2^)	2-h before	2.5±0.5	2.4±0.5	−9.6;±31.9	0.22
	1-h before	2.5±0.5	2.3±0.5	−14.4;±41.3	0.40
	Flight	2.6±0.5	2.1±0.4^a^^,^^b^	−26.2;±35.9	0.94
	1-h after	2.5±0.4	2.4±0.4	−11.2;±28.7	0.43
	2-h after	2.6±0.5	2.4±0.5	−11.6;±40.9	0.22

*HR, heart rate; RMSSD, root mean square of successive differences between normal sinus R-R intervals; SDNN, standard deviation of all R-R intervals; LnLF, natural log of the Low Frequency component; LnHF, natural log of the High Frequency; %Δ, percentage difference between days; CL, confidence limits; ES, effect size*;

a *, p < 0.05 vs. Control day*;

b *, p < 0.05 vs. other time points within the same day*.

**Table 4:** **Non-linear HRV measures (~1-h recordings) before, during, and after the flight on the experimental day and at matched times on the control day**.

	**Time**	**Control day**	**Experimental day**	**Magnitude of changes between days**	
				**%Δ; ± 90% CL**	**ES**
SD1 (ms)	2-h before	29.8±17.3	25.1±14.6	−15.7;±5.7	0.29
	1-h before	31.1±22.1	23.6±15.4	−24.1;±6.8	0.39
	Flight	31.7±18.0	17.6±10.6^a^^,^^b^	−44.4;±5.3	0.95
	1-h after	29.5±15.1	23.5±11.7	−20.3;±4.8	0.44
	2-h after	32.6±18.1	26.0±14.4	−20.2;±5.8	0.39
SD2 (ms)	2-h before	107.8±39.6	100.7±34.3	−6.5;±14.3	0.19
	1-h before	106.8±28.5	103.1±35.5	−3.3;±12.5	0.11
	Flight	111.7±47.4	101.4±37.8	−9.2;±16.5	0.24
	1-h after	115.4±60.9	103.3±37.9	−10.5;±19.4	0.23
	2-h after	122.6±50.4	108.8±42.1	−11.2;±17.9	0.29
SampEn	2-h before	1.01±0.28	0.86±0.27	−14.74;±0.1	0.54
	1-h before	1.03±0.29	0.89±0.21^a^	−13.52;±0.09	0.55
	Flight	1.06±0.34	0.79±0.34^a^^,^^b^	−26.62;±0.12	0.79
	1-h after	1.06±0.33	0.94±0.29	−11.19;±0.11	0.38
	2-h after	1.00±0.31	0.87±0.31	−12.71;±0.11	0.20
α1	2-h before	1.34±0.2	1.36±0.2	1.49;±0.09	0.10
	1-h before	1.33±0.2	1.39±0.2	4.51;±0.09	0.29
	Flight	1.29±0.2	1.48±0.2^a^^,^^b^	14.7;±0.09	0.94
	1-h after	1.29±0.2	1.37±0.2	6.20;±0.09	0.40
	2-h after	1.29±0.2	1.38±0.2	6.97;±0.09	0.44
α2	2-h before	0.99±0.12	0.97±0.11	−2.02;±0.04	0.10
	1-h before	0.97±0.14	0.96±0.10	−1.03;±0.04	0.29
	Flight	0.99±0.13	0.94±0.12	−5.05;±0.04	0.94
	1-h after	0.97±0.15	0.88±0.11^a^	−9.27;±0.04	0.40
	2-h after	1.00±0.17	0.93±0.11	−7.00;±0.05	0.44

*SD1, short-term beat-to-beat R-R variability from the Poincaré plot; SD2, long-term beat-to-beat variability from the Poincaré plot; SampEn, sample entropy; α1, detrended fluctuations of short-term fractal scaling; α2, detrended fluctuations of long-term fractal scaling;%Δ, percentage difference between days; CL, confidence limits; ES, effect size*;

a *, Different from Control day (p < 0.05)*;

b *, Different from both other time conditions within the same day (p < 0.05)*.

Compared to the Control day, SampEn was reduced in the hour prior to the flight (>13%, ES = 0.55) while α2 was reduced in the hour after the flight (>9%, ES = 0.40) (Table 4). During the flight, SD1 and SampEn were significantly lower (>26%, ES = 0.79–0.95) while α1 was significantly greater (>14%, ES = 0.94) compared to the control day (Table 4). All other variables were similar between days and times/moments (Table 4).

Values of 24-h SBP, DBP, MBP, and RPP on the control and experimental day are shown in Table 5. All BP derived measures were not significantly different between days although a small ES (ES = 0.38) for RPP was evident. Hour-by-hour analyses of these BP values before, during and after flights for both days are shown in Table 6. Mostly, there were significant increases for all variables during and after flights (ES = 0.54–1.43, Table 6) with an anticipatory effect only for SBP and RPP (ES = 0.45–0.75, Table 6).

**Table 5:** **Twenty-four hours blood pressure measures on the control and experimental days**.

			**Magnitude of changes between days**		
	**Control day**	**Experimental day**	**p**	**Δ%;%90 CL**	**ES**
SBP (mmHg)	107±5	107±3	0.60	4.40;±1.66	0.15
DBP (mmHg)	68±4	69±2	0.10	3.10;±1.17	0.19
MBP (mmHg)	81±2	82±2	0.21	3.40;±1.28	0.17
RPP (mmHg•bpm)	7881±1239	8345±1314	0.10	16.25;±496.9	0.38

*SBP, systolic blood pressure; DBP, diastolic blood pressure; MBP, mean blood pressure; RPP, rate pressure product; Δ%, percentage difference between days; CL, confidence limits; ES, effect size*.

**Table 6:** **Blood pressure measures before, during and after the flight on the experimental day and at matched times on the control day**.

	**Time**	**Control day**	**Experimental day**	**Magnitude of changes between days**	
				**%Δ; ± 90% CL**	**ES**
SBP (mmHg)	2-h before	106±4	109±6	2.33;±7.74	0.43
	1-h before	106±5	110±5^a^	4.05;±8.04	0.75
	Flight	107±4	113±8^a^	6.10;±7.09	0.99
	1-h after	106±4	110±7^a^	4.21;±6.71	0.71
	2-h after	104±5	109±5^a^	4.01;±4.43	0.79
DBP (mmHg)	2-h before	69±4	69±4	1.03;±11.05	0.07
	1-h before	69±5	70±6	2.45;±12.50	0.21
	Flight	67±5	71±7^a^	7.12;±15.39	0.69
	1-h after	66±2	71±5^a^	6.86;±9.77	1.08
	2-h after	65±3	71±5^a^	10.45;±10.80	1.43
MBP (mmHg)	2-h before	81±4	81±3	0.00;±7.46	0.06
	1-h before	81±4	83±5	2.06;±8.90	0.29
	Flight	80±4	84±6^a^	5.65;±10.65	0.77
	1-h after	79±2	83±5^a^	4.82;±7.15	0.92
	2-h after	78±3	84±5^a^	7.54;±7.02	1.31
RPP (mmHg•bpm)	2-h before	8475±1399	9149±1898^a^	8.96;±19.93	0.41
	1-h before	8362±1978	9160±1623^a^	15.37;±36.66	0.45
	Flight	8003±1416	10234±2249^a^	31.25;±35.90	1.22
	1-h after	8097±1924	9221±2313^a^	16.36;±25.92	0.54
	2-h after	7691±1947	9048±2002^a^	21.32;±25.86	0.70

*SBP, systolic blood pressure; DBP, diastolic blood pressure; MBP, mean blood pressure; RPP, rate pressure product; Δ%, percentage difference between days; CL, confidence limits; ES, effect size*;

a *, different from control day (p < 0.05)*.

Correlations between anthropometric, physical fitness and absolute values and relative changes (Δ%) in 24-h HRV measures are shown in Tables 7, 8, respectively. Absolute values of HRV measures during the experimental day only were significantly correlated with measures of aerobic fitness (positive relationships) and body composition (negative relationships, Table 7). Relative changes in HRV measures were significantly correlated with measures of aerobic fitness (negative relationships) and body composition (positive relationships, Table 8). There were no correlations identified between 24-h BP measures and physical fitness indices. Finally, significant and high correlations were observed between indices of aerobic fitness and body composition (*r* = −0.725 to −0.861; *p* < 0.01).

**Table 7:** **Correlation coefficients (***p***-value) between physical and fitness variables, and absolute values of 24-h HRV measures obtained on the Control and Experimental days**.

	**SDNN**	**RMSSD**	**LnLF**	**LnHF**	**SD1**	**SD2**	**SampEn**
**CONTROL DAY**							
BMI	−0.17 (0.43)	−0.30 (0.17)	−0.29 (0.20)	−0.16 (0.52)	−0.29 (0.19)	−0.16 (0.47)	0.13 (0.55)
MAS	0.02 (0.94)	0.09 (0.70)	0.06 (0.79)	0.14 (0.58)	0.10 (0.66)	0.03 (0.88)	−0.08 (0.72)
T_UMTT_	0.03 (0.88)	0.07 (0.75)	0.05 (0.82)	0.11 (0.65)	0.08 (0.71)	0.05 (0.82)	−0.06 (0.78)
BF	−0.10 (0.69)	−0.20 (0.37)	−0.12 (0.59)	−0.16 (0.52)	−0.20 (0.36)	−0.07 (0.74)	0.09 (0.68)
MS	−0.15 (0.51)	−0.03 (0.90)	−0.27 (0.23)	−0.32 (0.18)	−0.05 (0.84)	−0.16 (0.48)	−0.24(0.27)
**EXPERIMENTAL DAY**							
BMI	−0.49 (0.01)	−0.50 (0.01)	−0.63 (0.00)	−0.44 (0.04)	−0.50 (0.01)	−0.49 (0.03)	0.26 (0.24)
MAS	0.32 (0.14)	0.34 (0.12)	0.47 (0.03)	0.41 (0.06)	0.33 (0.13)	0.32 (0.15)	−0.02 (0.92)
T_UMTT_	0.35 (0.11)	0.36 (0.10)	0.51 (0.02)	0.43 (0.04)	0.35 (0.11)	0.35 (0.11)	−0.01 (0.97)
BF	−0.35 (0.11)	−0.43 (0.04)	−0.52 (0.02)	−0.44 (0.04)	−0.43 (0.04)	−0.34 (0.12)	0.17 (0.45)
MS	−0.15 (0.50)	−0.15 (0.51)	0.09 (0.68)	−0.12 (0.59)	−0.15 (0.51)	−0.15 (0.50)	−0.44 (0.04)

*BMI, body mass index; MAS, maximal aerobic speed; T_UMTT_, Time to complete the Université de Montreal Track Test; BF, body fat; MS, muscular strength; SDNN, standard deviation of all R-R intervals; RMSSD, root mean square of successive differences between normal sinus R-R intervals LnLF, natural log of the Low Frequency component; LnHF, natural log of the High Frequency component; SD1, short-term beat-to-beat R-R variability from the Poincaré plot; SD2, long-term beat-to-beat variability from the Poincaré plot; SampEn, sample entropy*.

**Table 8:** **Correlation coefficients (***p***-value) between changes (Δ%) in 24-h HRV parameters (control vs. experimental day) and physical fitness variables**.

	**ΔSDNN**	**ΔRMSSD**	**ΔLnLF**	**ΔLnHF**	**ΔSD1**	**ΔSD2**	**ΔSampEn**
BMI	0.59 (0.00)	0.61 (0.00)	0.54 (0.02)	0.53 (0.02)	0.61 (0.00)	0.59 (0.00)	−0.19 (0.39)
MAS	−0.27 (0.22)	−0.32 (0.14)	−0.031 (0.17)	−0.53 (0.02)	−0.32 (0.14)	−0.26 (0.24)	−0.02 (0.92)
T_UMTT_	−0.28 (0.21)	−0.32 (0.14)	−0.32 (0.17)	−0.52 (0.02)	−0.33 (0.13)	−0.27 (0.23)	−0.03 (0.91)
BF	0.39 (0.07)	0.48 (0.02)	0.34 (0.14)	0.54 (0.02)	0.49 (0.02)	0.37 (0.09)	−0.12 (0.59)
MS	−0.00 (0.99)	0.10 (0.69)	−0.34 (0.14)	−0.30 (0.23)	0.07 (0.74)	−0.01 (0.96)	0.32 (0.14)

*BMI, body mass index; MAS, maximal aerobic speed; T_UMTT_, Time to complete the Université de Montreal Track Test; BF, body fat; MS, muscular strength; SDNN, standard deviation of all R-R intervals; RMSSD, root mean square of successive differences between normal sinus R-R intervals LnLF, natural log of the Low Frequency component; LnHF, natural log of the High Frequency component; SD1, short-term beat-to-beat R-R variability from the Poincaré plot; SD2, long-term beat-to-beat variability from the Poincaré plot; SampEn, sample entropy*.

## Discussion

To our knowledge, this is the first study relating physical fitness, body composition and hemodynamic responses to flight induced stress (~1 h) in physically active individuals. The main findings were that a 1-h commercial flight was associated with enhanced cardiovascular stress (i.e., enhanced HR, SBP, DBP, MBP, and RPP) with vagal withdrawal and reduction in HR complexity, as indicated by the change in several HRV indices (i.e., RMSSD, LnHF, SD1, SampEn, α2). Moreover, greater aerobic fitness was positively associated with higher HRV during the experimental day (i.e., LnLF, LnHF) and less HRV change during a commercial flight day. Similarly, significant inverse relationships were identified between measures of body composition (% body fat and BMI) and HRV (RMSSD, LnLF, LnHF, SD1) during the experimental day with greater body fat associated with greater HRV changes during a commercial flight day. This study demonstrated that short-term commercial flying significantly altered cardiovascular function with greater physical fitness (e.g., greater aerobic capacity) and lower body fat composition associated with greater cardiovascular control (HRV and BP) for passengers during flights. Enhanced physical fitness and better body composition may enable passengers to cope better with the cardiovascular stresses associated with air travel for enhanced passenger well-being.

Compared to the control day, the 24-h HRV recordings on the experimental day indicated a significant increase in HR with reductions only in LnHF and α2 with the other HRV measures remaining relatively stable. Interestingly, when shorter periods of HRV were analyzed (i.e., hour to hour), flight-related stress was accompanied by a vagal withdrawal within the hour prior to take-off and was maintained for up to 2 h after destination arrival. This reduction in HRV with flight stress was previously noted in experienced (Oliveira-Silva and Boullosa, 2015) and novice military pilots (Sauvet et al., 2009) and reinforces that anticipatory autonomic responses occur prior to an announced stress (Hynynen et al., 2009; Boullosa et al., 2012). It is well known that adaptation in the face of stress is a major priority for all organisms (Ulrich-Lai and Herman, 2009) with an autonomic anticipatory change occurring in response to an expected biological stress (Boullosa et al., 2012). As the participants of the present study were passengers that had accumulated < 50 flying hours on commercial airlines, the anticipatory response could have been associated with flight inexperience (Waterhouse et al., 2002; Hynynen et al., 2009). Moreover, it has been shown that in experienced individuals (>500 h of flight), this anticipatory response is overridden (Oliveira-Silva and Boullosa, 2015). Therefore, it is likely that the observed vagal withdrawal of the current participants was associated with an acute, adaptive response of the ANS to cope with the upcoming stress. Likewise, it has been suggested that changes in the environment and daily routine could also contribute to a higher HR that would affect HRV (Zhao et al., 2015). Consequently, both anticipation of flight and changes in daily living conditions could modulate the cardiac autonomic responses as a biological preparatory mechanism for dealing with unusual and changing situations.

The observed greater 24-h HR during the experimental day was in agreement with previous studies (Sauvet et al., 2009) with stresses of multiple origin reported to increase HR (Kenefick et al., 2008; Boullosa et al., 2012). Despite the greater HR during the flight day, other 24-h cardiovascular measures (e.g., SBP, DBP, MBP, and RPP) were similar between days with this long-term assessment likely to be insensitive to acute flight stress. Importantly, short–term, 1-h measures were able to clearly identify the acute stress prior to, during and post-flight with significant changes and moderate to large effects (ES). Taken together, our results demonstrated that a commercial flight of only 1 h exposed travelers to a high allostatic load (McEwen and Seeman, 1999; Tonello et al., 2014), which exerted considerable neural and cardiovascular stress (Brundrett, 2001; Fowler et al., 2015). Furthermore, these stress-induced responses were capable of inducing alterations in the circadian rhythms such as blood pressure (Hansen et al., 2010), that can be maintained up to 2 h after the flight. It is important to note that these undesirable hemodynamic effects, although transient, may result in functional alterations in the hypothalamic-pituitary-adrenal axis (HPA) and hippocampus (Frodl and O'Keane, 2013), and negative influences on health in the long-term. Subsequently, attenuation of these transient changes associated with flight would be beneficial in reducing the negative impact of flight on the health of flyers. Generation of greater physical fitness may be an important means to modulate these flight-induced transient changes (Huang et al., 2013).

The relationship between indices of HRV and physical fitness was not surprising (Fronchetti et al., 2006; Hautala et al., 2010; Dutra et al., 2013; D'Agosto et al., 2014; Dolezal et al., 2014). However, to date there has been no consensus regarding the impact of the different components of physical fitness on HRV modulation during stress responses (Hautala et al., 2010; Bravi et al., 2013). We identified significant relationships between high HRV (absolute and change) and high aerobic fitness (MAS), and low % body fat. The current correlations between % body fat and HRV indices, specifically SD1 and RMSSD, were in line with the results of Yi et al. (2013) who noted that % body fat was inversely associated with several HRV measures after adjusting for age, gender, and cardiovascular risk factors. More recently, Tian et al. (2015) reported an inverse relationship between % body fat and HRV, and demonstrated that the decrease in % body fat was associated with an increase in HRV. Given that SD1 and RMSSD reflect parasympathetic activity (Fronchetti et al., 2006), the negative correlation between HRV and % body fat highlights the importance of maintaining desirable levels of % body fat (Pillay et al., 2015) to optimize HRV for stress management. The incorporation of physical exercise has been well documented as an effective means to manage % body fat (Golubic et al., 2015) as well as providing an important intervention to moderate stress-related autonomic modulation (Dolezal et al., 2014). In fact, we observed significant correlations between measures of aerobic capacity (MAS and T_UMTT_) with ΔLnHF (*r* = −0.53 and *r* = −0.52, respectively) that highlights physical fitness as an important modulator.

In line with the previous discussion, it is worthy to note that the current participants were healthy and physically active men with good levels of physical fitness (Garber et al., 2011). Despite this degree of physical fitness, the current participants still experienced flight-induced stress with their low % body fat and high VO_2_max likely providing a degree of protection against stressful situations. It remains to be seen whether less fit individuals can cope with flight-induced stress in the same manner or whether they exhibit substantially greater stress and cardiac autonomic disturbances. Meanwhile, given that aerobic fitness and body composition measures were also correlated between each other, further studies should clarify the relative influence of these health related components on cardiovascular responses to commercial flights, with special attention to differences between evaluation protocols (e.g., Treadmill vs. Cycle vs. Field running) and selected parameters (e.g., actual VO_2_max vs. Estimated VO_2_max).

Finally, it should be pointed out that muscular strength was not significantly correlated with HRV indices, except for SampEn (*r* = −0.44; *p* = 0.04), a contrary finding to that of Heffernan et al. (2007) who demonstrated that a program of resistance exercises enhanced SampEn in young healthy men. An explanation for this lack of association was not apparent in the current study however, others (Barbosa et al., 2014) have demonstrated that resistance training could positively alter HRV of healthy men and hypertensive women. Therefore, we suggest that enhancement of muscular strength through resistance training should not be disregarded as a strategy to improve cardiac autonomic modulation as it has been shown to be of great value for HRV and performance (Caruso et al., 2015). Further studies are encouraged to examine the role of resistance exercise for cardiac autonomic control and coping with stress.

In summary, the results of the present study partially confirmed our hypothesis that specific aspects of physical fitness (e.g., aerobic capacity) were related to better cardiovascular control (HRV and BP) for passengers during flights. Further, these results reinforced the need for future studies to investigate the role of physical fitness in reducing the flight-induced stress and related cardiac autonomic alterations for the general population. Current recommendations for air travel such as alcohol and tobacco abstention (Mills and Harding, 1983; Newman, 2004), maintenance of adequate hydration (Meir et al., 2003) and careful activity planning on destination arrival (Reilly et al., 2009) may assist with reductions in flight-induced stress and allostatic load that remain to be investigated. Finally, our results indicate that persons who frequently fly should develop and maintain suitable levels of physical fitness to reduce the potential impact of exposure to the cardiovascular stress associated with allostatic loads during air travel.

## Author contributions

Study Design: IO, DB. Data collection: IO, Data analyses: IO, Interpretation of the results: IO, AL, MM, HS, SR, CC, DB. Manuscript writing: IO, AL, MM, HS, SR, CC, DB. Approved the final manuscript version: IO, AL, MM, HS, SR, CC, DB.

## Funding

DB, HS enjoy a productivity grant in research from CNPq.

## Conflict of interest statement

The authors declare that the research was conducted in the absence of any commercial or financial relationships that could be construed as a potential conflict of interest. The reviewer BM and handling Editor declared their shared affiliation, and the handling Editor states that the process nevertheless met the standards of a fair and objective review.
